# Caesarean Section Scar and Placental Location at the First Trimester of Pregnancy—A Prospective Longitudinal Study

**DOI:** 10.3390/medicina60050719

**Published:** 2024-04-26

**Authors:** Egle Savukyne, Mindaugas Kliucinskas, Laura Malakauskiene, Kristina Berskiene

**Affiliations:** 1 Department of Obstetrics and Gynaecology, Medicine Academy, Lithuanian University of Health Sciences, 44307 Kaunas, Lithuania; mindaugas.kliucinskas@lsmu.lt (M.K.); laura.malakauskiene@lsmu.lt (L.M.); 2 Department of Sports Medicine, Lithuanian University of Health Sciences, Tilzes Street’ 18, 47181 Kaunas, Lithuania; kristina.berskiene@lsmu.lt

**Keywords:** caesarean section, placental location, ultrasonography, first trimester, scar niche

## Abstract

*Background and Objectives*: This study aims to report the location of the placenta in the first trimester of pregnancy in groups of women according to the number of previous caesarean deliveries and the visibility of the caesarean scar niche. *Materials and Methods*: The prospective observational research included adult women aged 18 to 41 years during pregnancy after one or more previous caesarean sections (CSs). Transvaginal (TVS) and transabdominal sonography (TAS) was used to examine the uterine scar and placental location during 11–14 weeks. The CS scar niche (“defect”) was bordered in the sagittal plane as a notch at the previous CS scar’s site with a depth of 2.0 mm or more. A comparative analysis of the placental location (high or low and anterior or posterior) was performed between groups of women according to the CS number and the CS scar niche. *Results*: A total of 122 participants were enrolled during the first-trimester screening. The CS scar defect (“niche”) was visible in 40.2% of cases. In cases after one previous CS, the placenta was low in the uterine cavity (anterior or posterior) at 77.4%, and after two or more CSs, it was at 67.9%. Comparing the two groups according to the CS scar niche, the placenta was low in 75.5% of cases in the participant group with a CS scar niche and in 75% of cases without a CS scar niche (*p* = 0.949). *Conclusions*: The number of previous caesarean deliveries has no effect on the incidence rate of low-lying placentas in the first trimester. Moreover, the presence of the CS scar niche is not associated with anterior low-lying placentas.

## 1. Introduction

The evolution of the placenta is understood as a complex process. The placental location is important for developing a satisfactory foetal–maternal interface. However, it is not clear how the uterine scar or scar niche after a previous caesarean section (CS) affects the placental location site in the uterus interface.

Low-lying placentas, known as praevia and placenta accreta spectrum disorders, are the most frequent placental abnormalities. These placental abnormalities can have severe obstetric complications because of extreme blood loss during or after delivery [1]. The aetiology of these placental abnormalities needs to be better understood, as their incidence is increasing because of the rising caesarean delivery rates. The incidence of placenta praevia is increasing due to a previous caesarean section and in vitro fertilisation [2]. The term placenta praevia describes when the placenta covers the internal cervical os. The prevalence of placenta praevia changes significantly with gestational age because of “placental migration” [3]. Placenta praevia or low-lying placenta is more commonly seen in early gestation and is present in up to 6.2% of pregnancies between 12 and 16 weeks of gestation than at term, where the rate is only 0.16% [4]. The uterine wall grows faster during pregnancy than the placenta-covered uterine wall areas [5]. The “placental migration” process fits in the intact uterus but not in a uterus with a CS scar [6]. The frequency of insufficient scars increases with the number of CSs and is influenced by the uterine closure technique and previous scar location in the uterus [7,8]. The low uterine wall segment becomes thinner after repeated CSs and contains more fibrotic tissue [9].

A CS scar may affect myometrial contractility, disturbing uterine contraction waves at implantation. In cases after caesarean delivery, the placenta is more likely located at the posterior side of the uterus [6]. Moreover, implantation may occur in the CS scar niche area because proteins responsible for normal endometrial receptivity during implantation (integrin β3 and leukaemia inhibitory factor) are too expressed in the scar area. This mechanism can cause implantation around the uterine scar [10]. Also, hypoxia in the CS scar area stimulates trophoblast cells to proliferate and embryos can develop here [11]. It is known that the fundus area in the uterus has the best endometrial blood flow and is most suitable for implantation [12]. A total of 76% of embryos migrate towards the fundus, and some migrate to the cervix (11%) [13,14].

Nevertheless, according to current first-trimester ultrasound guidelines, it is not recommended to report the presence of a placenta praevia between 11^+0^ and 13^+6^ weeks of gestation [15]. Placenta praevia at term can cause life-threatening bleeding, requiring a hysterectomy, blood transfusion, intensive care, or even leading to maternal death [16]. According to the massive risk of antenatal complications for mothers with placenta praevia (anterior vs. posterior), it is a very important to identify it and take preoperative planning steps (preparation of blood products and potential surgical procedures). The relationship between the CS scar niche and the placental site in a subsequent pregnancy has not been previously assessed.

Ultrasonography is the best modality for assessing the placenta in the first trimester of pregnancy [4]. The CS scar is known as a definite risk factor for abnormal placentation. The CS scar niche is mainly evaluated using transvaginal sonography (TVS) as the placental location could be examined in both transvaginal and transabdominal approaches. Identifying the location of the placenta in the first trimester of pregnancy is not as easy because of uterine flexion and extension. To improve the accuracy of the placental site in the first trimester, an ultrasound is recommended to identify the cervix and the anterior and posterior uterine walls.

The study aimed to evaluate how several previous caesarean deliveries and a CS scar niche can influence the placenta’s site during the first trimester of pregnancy. We also tried to assess whether or not specific placental locations affect the placenta’s characteristics and pregnancy outcomes.

## 2. Materials and Methods

**Figure 1:**
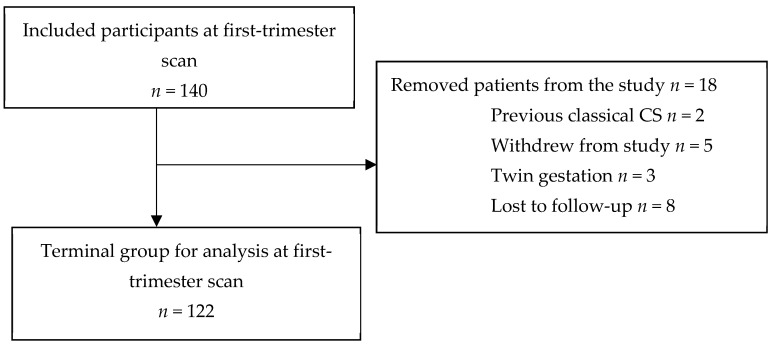
Diagram of inclusion of patients in the study.

This prospective investigation was performed at the Hospital of Lithuanian University of Health Sciences Kaunas Clinics, Lithuania, by approval from Kaunas regional bioethics committee (protocol no. BE-10-15) [17]. The trial was registered on 18 March 2019 (Australian New Zealand Clinical Trials Registry, No. ACTRN12619000435189 (http://www.ANZCTR.org.au/ACTRN12619000435189.aspx, accessed on 18 March 2019). Between March 2019 and October 2020, 140 patients were included. All participants signed an informed consent form. We included women over 18 years with a singleton pregnancy after one or more previous low-transverse CSs. The datasets used during the study are accessible from the corresponding author upon reasonable request. Three women with twin gestations were excluded from the study, and two women were removed after previous classical CS. Eight participants were lost to follow-up; five patients were removed from the study after the first-trimester ultrasound scan, and, in the end, there were 122 patients (Figure 1) [17].

The patient’s past obstetric history was documented, including previous CSs and demographic data, such as age and body mass index (BMI). The first sonographer (ES) with experience in performing cervix and CS scar ultrasounds performed all examinations with Voluson E8 Expert (GE Healthcare Korea Co., Ltd., Seoul, Republic of Korea) systems using a 5–9 MHz transvaginal probe and a 4–5 MHz transabdominal probe. The second sonographer (LM) was invited only for 24 non-selected cases to evaluate the placental site at the first-trimester screening. All ultrasound scans for uterine scar evaluation and placental location were carried out in the lithotomy position. CS scar evaluation was performed in a standard midsagittal view of the uterus isthmus and internal cervical os using a transvaginal probe. The CS scar niche or defect was defined when there was a hypoechoic area of the myometrium in the lower uterine segment at the site of the previous CS. The study participants were subjectively allocated to visible CS scars and non-visible CS scars. Then, visible scars were classified as forming or not forming a CS scar niche (the depth of the low echogenicity part was at least 2.0 mm).

**Figure 2:**
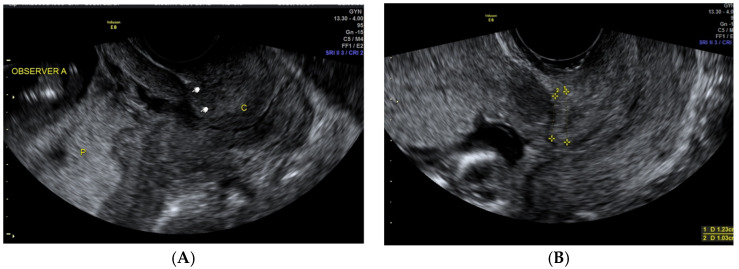
(**A**) TVS at the first trimester of pregnancy, posterior-low placenta and CS scar niche. P, placenta; C, cervix. (**B**) TVS at the first trimester of pregnancy, visible CS scar, non-forming niche, anterior-low placenta.

The placental site was evaluated with a transabdominal probe. The women were asked to have their bladder filled normally during the ultrasound examination. In cases where the transabdominal ultrasound indicated a low-lying placenta, a transvaginal scan was applied. Placental locations were recorded prospectively using high-anterior, high-posterior, low-anterior, and low-posterior subgroups. In cases when the placenta was “left- or right-anterior”, it was classified as “anterior”, and similarly, “right- or left-posterior” was classified as only “posterior” (Figure 2A,B).

Twenty-four unselected continuous cases were allocated to test an interobserver agreement for allocating the placental site in the uterus. Both sonographers (ES and LM) used real-time scanning to assess the placental location and visualize a CS scar niche at the first-trimester screening ultrasound examination. At each examination, the first sonographer (ES) estimated the placental site in both sagittal and transverse planes, and the results were documented in a table. Another sonographer (LM) examined the same patient, without viewing the results of the first sonographer, and reperformed the examination. Management of the patients was carried out according to the local hospital’s policy. As a part of routine clinical practice, patients with low-lying placentas in the first trimester of pregnancy scan underwent an ultrasound evaluation at 18–20 and 34 weeks gestation.

Results were recorded prospectively on an SPSS spreadsheet, and illustrative images of the placental locations and uterine scars were saved on the hospital image storage system (DICOM). Pregnancy outcomes and complications were extracted from the hospital archives after the patient’s delivery.

The sample volume and statistical analysis were calculated using SPSS Statistics v 27.0 program (IBM Corp., Armonk, NY, USA). The Chi-square test was used to determine differences in categorical variables, or in the case of insufficient cases in the categories, Fisher’s exact test was used. The distribution of quantitative variables to normal variables was compared in independent groups (after one CS versus after two or more CSs, CS scar niche group versus non-niche group) using the Mann–Whitney U-test. A value of *p* < 0.05 was considered statistically significant.

## 3. Results

A total of 122 participants were admitted to a prospective study to assess the placental site in the uterus and CS scar visibility from the first trimester of pregnancy. A considerable number of the women had one previous CS 94/122 (77.4%), and 26 women had two previous CSs (21.3%). Only two participants had three previous CSs (1.6%). The age of the participants ranged from 22 to 41, and their BMIs differed from 17 to 36 kg/m^2^. Most participants 56 (45%) had an average body weight (18.5–24.9 kg/m^2^); 46 (37.7%) were overweight (25.0–29.9 kg/m^2^); 15 (12.3%) had first-degree obesity (30.0–34.9 kg/m^2^); and 4 (3.3%) had second-degree obesity (35.0–39.9 kg/m^2^). The number of births differed from two to five in all the study groups. Labour dystocia was the most frequent indication for the first caesarean delivery, with numbers reaching 63 (55.8%) cases. A total of 26 (23%) caesarean deliveries were due to poor foetal condition during delivery, and 24 caesarean deliveries (21.2%) were due to breech presentation. 

The CS scar was evident on an ultrasound in 95/122 (77.9%) of cases, and the scar niche was found in 49/122 (40.2%) of all study groups. Half of those with visible CS scars had a CS scar niche 49/95 (51.5%). The CS scar formed a niche in women after one CS and after two or more previous CSs without statistical differences (43.6% vs. 28.5%; *p* = 0.228). On the other hand, non-visible CS scars were more frequent after one CS than two or more previous CSs (63% vs. 37%; *p* = 0.049).

We investigated the influence of maternal and obstetric history on the placenta location in the following pregnancy. Uterine abrasion significantly influenced scar niche development (*p* = 0.049) but did not statistically impact the low-lying placenta (Table 1 and Table 2).

In contrast, no statistically reliable differences were established between the scar niche and maternal age, smoking status, BMI, gestational diabetes, and vaginal birth in the past (Table 2). Women with low-lying placentas were significantly older (*p* = 0.016) than those with high-located placentas. On the contrary, the BMI was not statistically reliable with any definitive placental location (*p* = 0.189) (Table 2).

The most common location of the placenta in the study population at the first-trimester ultrasound examination was posterior low-lying 48/122 (39.7%) (95% CI:31.4–48.8); *p* < 0.05). Our study results show that a low-lying placenta (anterior and posterior) was found in 91/122 (75.2%) cases, and in 31/122 (24.8%) cases, the placenta was located high in the uterus (anterior or posterior). According to the location in the uterus, 88/91 patients (96.7%) with low-lying placentas go through migration in the second–third trimester of pregnancy and develop into normal placentas. Agreement on the placental location between the two sonographers was obtained in 23/24 cases (95.8%), and overall agreement on the visibility of the uterine scar at the first-trimester scan was 100% (*p* = 0.002).

Comparing the prevalence of the placental location between the groups of women, a low-lying placenta occurred with the same frequency in the CS scar niche group as in the no-niche group without any differences (75.5% vs. 75% (OR = 0.973 (95% CI: 0.419–2.258); *p* = 0.949)) (Table 3).

The number of previous CSs did not affect the placental location (Table 4). In the group after one CS, the placenta was located low in 72/93 (77.4%) cases, although in the group after two or more CSs—19/28 (67.9%) cases (OR = 0.616; (95% CI: 0.24–1.565).

A total of 63 women from the study group underwent a trial of labour after one previous CS, and 41/63 (65.0%) underwent a successful vaginal delivery. A total of 22/63 (35.0%) of the participants underwent an emergency repeat CS after a trial of labour. From the group of women that underwent a trial of labour after one previous CS, 27/63 (42.8%) of the participants were with labour induction, and from them, 15/27 (55.5%) had a successful vaginal delivery. The mean gestational age at delivery was 38.8 ± 2.37 weeks, with a neonatal weight of 3473.7 ± 598.0 g. In two cases (3.2%), uterine dehiscence occurred, and both had confirmed CS niches on ultrasound examination [17]. There were no uterine ruptures in the study population. Placenta praevia were diagnosed in three participants (2.4%). One case was diagnosed as placenta praevia increta (0.8%) for women after two previous CSs, and two (1.6%) cases of placenta accreta with placenta praevia partialis. During a first-trimester ultrasound examination, the placenta was low-lying on the anterior wall in all three cases.

## 4. Discussion

We have shown that the number of previous caesarean deliveries does not affect the placental location in the uterus as it is not a caesarean scar niche. Nevertheless, low-posterior placentas occurred frequently in our study population. The history of CS is more likely to be a risk factor for placental location on the posterior wall in a subsequent pregnancy. This agrees with a previous study by O. Naji [6]. They concluded that CS scars could be related to an increased risk for posterior placentas, which are low numbers that implant in the fundus of the uterine cavity. This could be because of elevated numbers of pregnancy losses if the implantation occurs at the site of the CS scar and the anterior wall [6]. Concerning the other variables, we found that maternal age affected placental location. According to previous studies, older women have significantly more low-lying placentas [6,18]. 

We did not assess the statistical discrepancy in the prevalence of low-anterior or low-posterior placentas according to the number of previous CSs. However, this may have been due to the low number of participants after the previous two or more CSs. Our data also suggest that a CS scar has no significant effect on placental migration in the cases of a low-lying placenta at the beginning of pregnancy.

This investigation suggests that uterine scarring can influence implantation. Nonetheless, the consequence of this investigation needs to be clarified. There is growing confirmation that defective decidualisation causes abnormal placentation and its subsequent effects on pregnancy outcomes. Moreover, the scar niche might modify myometrial contractility, disrupting natural deflation waves of the endometrium [19,20].

A strength of this study is that it is prospective, as all scans were performed by one investigator (ES) according to standardised procedures using transabdominal and transvaginal ultrasonography to evaluate the placental site in the uterine cavity and the visibility of the uterine scar. The study design, with included cases after a previous transverse CS with singleton pregnancies, eradicates the potential effect of multiple pregnancies. To our accomplishments, the association between the placental site in the uterus and pregnancy outcomes in women with uterine scars has barely been studied.

The study’s main limitation is the limited number of participants and the very small number of placenta praevia at term. Also, a limitation is that ultrasound scans were performed in the first trimester of pregnancy when the placenta is known to subsequently migrate. However, we intended to review the placental site at the first-trimester ultrasound screening, as this is a routine scan performed for most pregnant women in Lithuania. Our research categorised the placental location into four locations, according to the cervix and bladder visualisations in the first trimester of pregnancy. This categorisation may need to be more precise. Nevertheless, data about placental location were registered prospectively in our study, and any misanalysis can be assumed to be non-differential. Further analysis with a control group without scars in the uterus is needed to understand the role of the CS scar “defect“ on the healing process and implantation as the location of the placenta in a subsequent pregnancy.

## 5. Conclusions

The presented study shows that women with uterine scars after a previous CS often have the placenta located on the posterior wall of the uterus, as older women more often have low-lying placentas in the first trimester. Nevertheless, the number of uterine scars do not affect low-lying placentas in the first trimester of pregnancy or at term.

## Figures and Tables

**Table 1:** Demographic factors and obstetric history about placental location in groups of women according to CS scar niche and several previous CSs.

Parameter	CS Niche *n* = 49 Median (IQR) or *n* (%)	No CS Niche *n* = 73 Median (IQR) or *n* (%)	*p*	1 CS *n* = 94 Median (IQR) or *n* (%)	2 or More CSs *n* = 28 Median (IQR) or *n* (%)	*p*
Age (years)	34.0 (29.5–36)	35.0 (29–27.0)	0.421	34.0 (29–36.0)	34.6 (33–37)	0.078
BMI (kg/m^2^)	24.9 (21.6–28.2)	25.5 (21.9–28.4)	0.529	25.4 (22–28.2)	25.1 (20.3–30.2)	0.956
Parity	3 (2–3)	3 (2–3)	0.80	2 (2–3)	3 (3–6)	0.80
Number of previous CSs						
1	34 (69.4)	45 (61.6)	0.455	N/A	N/A	N/A
2	13 (26.5)	26 (35.6)				
3	2 (4.1)	1 (1.4)				
4	0	1 (1.4)				
Previous uterine curettage	18 (36.7)	14 (19.2)	0.037	25 (26.6)	7 (25.0)	1.0
Smoker	5 (10.2)	11 (15.1)	0.586	11 (11.7)	5 (17.9)	0.523
Hypertension	7 (14.3)	5 (6.8)	0.220	10 (10.6)	2 (7.1)	0.732
Gestational diabetes	6 (12.2)	9 (12.3)	1.0	11 (11.7)	4 (14.3)	0.746
VBAC	3 (6.1)	5 (6.8)	1.0	7 (7.40	1 (3.6)	0.680
Eclampsia	3 (6.3)	3 (4.1)	0.681	6 (6.5)	0	0.224

BMI, body mass index; CS, caesarean section; VBAC, vaginal birth after caesarean delivery.

**Table 2:** Demographic aspects and obstetric history concerning placental location in the first trimester of pregnancy.

Parameter	High Placenta Median (IQR) or *n* (%)	Low Placenta Median (IQR) or *n* (%)	*p*
Age (years)	30.5 (28–35.2)	35 (30–37)	0.028 *
BMI (kg/m^2^)	26.1 (21.1–30.6)	25.3 (22.4–28)	0.338 *
Smoker	7 (43.8)	9 (56.3)	0.700 **
Visible CS scar	23 (24.5)	71 (75.5)	1.000 **
Non-visible CS scar	7 (25.9)	20 (74.1)	1.000 **
Uterine curettage	6 (18.8)	26 (81.3)	0.494 **

BMI, body mass index; CS, caesarean section; * Mann–Whitney test. ** Chi-square test.

**Table 3:** Location of the placenta in the first trimester of pregnancy in cases with and without CS scar niche.

Placental Location	CS Scar Niche Group *n* = 49 *n* (%)	No-Niche Group *n* = **73** *n* (%)	*p*
High (anterior or posterior)	12 (24.5)	18 (25)	0.949
Low (anterior or posterior)	37 (75.5)	54 (75)	0.949
Anterior low-lying	18 (60)	25 (58)	0.874
Posterior low-lying	19 (38.8)	29 (40.3)	0.972

CS, caesarean section.

**Table 4:** Placental location at the first trimester of pregnancy in the study groups after one previous CS and after two or more CSs.

Placental Location	One Previous CS *n* = 93 *n* (%)	Two or More Previous CSs *n* = 28 *n* (%)	*p*
High (anterior or posterior)	21 (22.6)	9 (32.1)	0.307
Low (anterior or posterior)	72 (77.4)	19 (67.8)	0.307
Anterior low-lying	35 (37.6)	8 (28.6)	0.516
Posterior low-lying	37 (39.8)	11 (39.3)	0.815

CS, caesarean section.

## Data Availability

The data given in this research are available on request from the corresponding author.
